# Aids to Nursing

**Published:** 1888-03-31

**Authors:** M. M. Basil

**Affiliations:** Author of "The Commoner Accidents to Life and Limb"


### Aids to Nursing.

By M. M. Basil, M.A., M.B., C.M., Author of "The Commoner Accidents to Life and Limb."

*(Continued from p. 426.)*

### FOOD AND FEEDING.

In cases of prostration feeding must be attended to during the night just as regularly as in the day. Nourishment must not be forgotten during the long sleep which follows several days of wakefulness and exhaustion. An inexperienced nurse is sometimes in doubt whether she should awaken the patient or not. Please remember to make inquiries in each case when you are in doubt on this point.

You must take precautions to prevent the patient dying in your hands during feeding. Even in cases where death is the only possible issue of the disease, such an accident is very disagreeable. It is not likely to be forgotten by the friends of the patient. The process of swallowing in weak patients, especially those suffering from diphtheria, laryngitis, and palsy, particularly bulbar paralysis and the general paralysis of the insane, must be observed; and, if on swallowing fluids, violent coughing or spluttering through the nose supervenes, you should withhold all solids from the patient and call the doctor's attention to the fact at the next visit. In patients who are deeply unconscious, weak, or paralysed, the process of swallowing must be induced by rubbing the gums before any large quantity of liquid is poured into the mouth.

The method of compelling a patient to swallow food by filling the back of the mouth with it and then holding the nose is a dangerous one, and should never be resorted to by a nurse. I do not think this caution uncalled for at this time of day, as I know of one case of death due to its nonobservance, where not one, but two nurses were responsible for the accident.

The selection of suitable diet for the patient is not a part of the nurse's duty; still, however, a few points connected with this subject must be taken into consideration. The sick should have no tea or coffee after five p.m., otherwise a sleepless night may be the consequence. In certain cases of nervous exhaustion, tea given late at night or even towards the morning may succeed in securing some hours' rest. Warm drinks, such as hot milk, warm water, porter, and porridge or toddy may act as good sleeping-draughts, if given at the right time. Sleeplessness arising from hunger is more common than is usually imagined.

Strong coffee, when ordered for medicinal purposes, must be made from the roasted berry. It is a medicine on which much reliance is placed in cases of opium poisoning. As generally used, it is a perfect sham. There is no ground coffee to be had in England. The State takes good care to provide against the existence of such luxuries.

In cases of vomiting, food must be given often and in small quantities. The liquids ordered may be administered cold or warm, according to indications. But here, as well as in diarrhœa and dysentery, iced milk or cold soups with no salt or pepper will usually suit best. It would be well to boil the milk and allow it to cool before using it in the sick room. Milk is in such constant use that it is worth your while to remember that a lactometer, constructed on exactly the same principle as the urinometer frequently employed in the wards, will enable you to know the genuine article from its adulterations. It has often been observed that the use of this simple instrument in every tenth house in England would sweep 75 per cent. of all cases of milk adulteration out of the country in a week. Along with the commonest of milk adulterations, viz., added water, would also disappear most of the milk diseases.

Very little salt must be put into the food if the patient's mouth is sore or the stomach irritable. It is often forgotten that salt is very irritating to a mouth which is practically wounded at a number of points. On the other hand, many convalescents who have a fierce craving for murderous articles of diet—such as cured fish, pork, etc.—ought to have their food very salt. The craving for such food arises mainly from the fact that these articles contain salt in abundance. It can therefore be appeased to a large extent by giving them salt *ad libitum*.

*(To be continued.)*

